# An MVA-based vector expressing cell-free ISG15 increases IFN-I production and improves HIV-1-specific CD8 T cell immune responses

**DOI:** 10.3389/fcimb.2023.1187193

**Published:** 2023-05-29

**Authors:** Michela Falqui, Beatriz Perdiguero, Rocio Coloma, Manuel Albert, Laura Marcos-Villar, Joseph Patrick McGrail, Carlos Óscar S. Sorzano, Mariano Esteban, Carmen Elena Gómez, Susana Guerra

**Affiliations:** ^1^ Department of Preventive Medicine, Public Health and Microbiology, Universidad Autónoma de Madrid, Madrid, Spain; ^2^ Department of Molecular and Cellular Biology, Centro Nacional de Biotecnología, Consejo Superior de Investigaciones Científicas (CSIC), Madrid, Spain; ^3^ Centro de Investigación Biomédica en Red de Enfermedades Infecciosas (CIBERINFEC), Instituto de Salud Carlos III (ISCIII), Madrid, Spain; ^4^ Biocomputing Unit and Computational Genomics, Centro Nacional de Biotecnología, Consejo Superior de Investigaciones Científicas (CSIC), Madrid, Spain

**Keywords:** ISG15, adjuvant, vaccine, inmunity, HIV, IFN, Modify Vaccinia virus Ankara

## Abstract

The *human immunodeficiency virus* (HIV), responsible of the Acquired Immune Deficiency Syndrome (AIDS), continues to be a major global public health issue with any cure or vaccine available. The *Interferon-stimulated gene 15* (ISG15) encodes a ubiquitin-like protein that is induced by interferons and plays a critical role in the immune response. ISG15 is a modifier protein that covalently binds to its targets via a reversible bond, a process known as ISGylation, which is the best-characterized activity of this protein to date. However, ISG15 can also interact with intracellular proteins via non-covalent binding or act as a cytokine in the extracellular space after secretion. In previous studies we proved the adjuvant effect of ISG15 when delivered by a DNA-vector in heterologous prime-boost combination with a Modified Vaccinia virus Ankara (MVA)-based recombinant virus expressing HIV-1 antigens Env/Gag-Pol-Nef (MVA-B). Here we extended these results evaluating the adjuvant effect of ISG15 when expressed by an MVA vector. For this, we generated and characterized two novel MVA recombinants expressing different forms of ISG15, the wild-type ISG15GG (able to perform ISGylation) or the mutated ISG15AA (unable to perform ISGylation). In mice immunized with the heterologous DNA prime/MVA boost regimen, the expression of the mutant ISG15AA from MVA-Δ3-ISG15AA vector in combination with MVA-B induced an increase in the magnitude and quality of HIV-1-specific CD8 T cells as well as in the levels of IFN-I released, providing a better immunostimulatory activity than the wild-type ISG15GG. Our results confirm the importance of ISG15 as an immune adjuvant in the vaccine field and highlights its role as a potential relevant component in HIV-1 immunization protocols.

## Introduction

The SARS-CoV-2 coronavirus pandemic and the new multi-country monkeypox outbreak in non-endemic regions highlight how essential is for the global public health the development of effective vaccines that protect people from viral infection outbreaks. Among the pandemics currently affecting the world, the acquired immunodeficiency syndrome (AIDS) caused by the *human immunodeficiency virus* (HIV) that began in 1981 (Barré-Sinoussi et al., 1983) is an ongoing major public issue. To date there is no functional cure or vaccine able to prevent HIV-1 infection, although anti-retroviral therapies (ART) have converted HIV in a chronic infection. Since the practice of immunization developed by Edward Jenner in 1796 against smallpox, our knowledge about the immune response and signaling pathways involved has rapidly evolved (Meyer et al., 2020). The need to improve the mode of action of current vaccines highlights the importance of adjuvants, especially in the HIV field (Rao and Alving, 2016). One patented^1^ promising adjuvant is the Interferon (IFN)-stimulated gene (ISG) 15 (ISG15) (Villarreal et al., 2015; Gómez et al., 2020).

ISG15 is one of the first discovered ISGs that could be induced even under other types of stimuli (Malakhova et al., 2002; Liu et al., 2004; Pitha-Rowe et al., 2004; Radoshevich et al., 2015). It is a small ubiquitin-like (Ubl) protein, composed by two ubiquitin-like domains linked by a hinge region (Narasimhan et al., 2005) with a central role in host antiviral response (Morales and Lenschow, 2013; Perng and Lenschow, 2018). ISG15 exists in different forms: intracellular unconjugated or conjugated to its target proteins, and in a soluble form which is released into extracellular medium. ISG15 is synthesized as a precursor of 17 kilodaltons (KDa) that is proteolytically cleaved into its mature 15 KDa form. This process exposes a carboxy-terminal LRLRGG motif that is essential for its covalent conjugation to target proteins through a three-step reversible process known as ISGylation (Loeb and Haas, 1992; Narasimhan et al., 1996). The functional effect of ISGylation is still poorly understood. One possibility is that ISGylation alters cellular localization and protein function. Moreover, in the case of proteins that can polymerize or multimerize, the modification of a small portion of the protein could impair the assembly of protein complexes (Albert et al., 2018). To date, an antiviral effect mediated by ISG15 or ISGylation has been described using *in vitro* and/or *in vivo* systems for many viruses (Morales and Lenschow, 2013), including *Influenza A virus* (IAV) (Tang et al., 2010; Zhao et al., 2010), *Respiratory syncytial virus* (RSV) (González-Sanz et al., 2016), HIV (Okumura et al., 2006) and Ebola virus (EBOV) (Okumura et al., 2008). Given the importance of the antiviral response governed by ISG15, it is not surprising that several viruses, such as *Influenza B virus* (IBV) (Zhao et al., 2016) and *severe acute respiratory syndrome coronavirus-2* (SARS-CoV-2) (Freitas et al., 2020; Shin et al., 2020; Liu et al., 2021; Munnur et al., 2021), have evolved strategies to counteract its antiviral effects (Guerra et al., 2008; Perng and Lenschow, 2018).

Despite the absence of a signal peptide (Knight and Cordova, 1991; D’Cunha et al., 1996b), ISG15 is released into the extracellular space by different mechanisms (Perng and Lenschow, 2018), exhibiting immunomodulatory activities (D’Cunha et al., 1996a; Swaim et al., 2017) in dendritic cells (DCs) maturation (Padovan et al., 2002), natural killer (NK) cells proliferation (Iglesias-Guimarais et al., 2020), regulation of Interferon-γ (IFN-γ) secretion (Bogunovic et al., 2012; Fan and Zhang, 2013) and as a chemotactic factor for neutrophils (Owhashi et al., 2003). The Lymphocyte function-associated antigen-1 (LFA-1) receptor has been identified as the cellular receptor for extracellular ISG15 in NK cells. This interaction leads to IFN-γ and Interleukine-10 (IL-10) release in IL-12-pretreated cells (Swaim et al., 2017). Furthermore, Narasimhan et al. highlighted the importance of two cysteine domains in the hinge region on ISG15 (Narasimhan et al., 2005), which are essential for dimer formation and for its cytokine activity, probably involved in the inflow of IL-1β–producing CD8αDCs to the site of infection (Napolitano et al., 2018).

According to previous studies, ISG15 can act as an adjuvant inducing an increase in the immune response mediated by cytotoxic T lymphocytes (CTLs), without interfering with the levels of CD4 lymphocytes, in both cancer (Villarreal et al., 2015; Chen et al., 2020) and therapeutic vaccine fields (Iglesias-Guimarais et al., 2020). In this sense, we have previously reported an increase in the HIV-1 Env-specific cellular immune response after the co-administration of DNA vectors expressing either the wild-type ISG15GG or the mutated ISG15AA forms with HIV-1 antigens in an heterologous prime/boost combination with the MVA-B vector (Gómez et al., 2020). MVA-B is an MVA-based recombinant virus that simultaneously expresses the monomeric HIV-1 BX08 gp120 protein as a cell-released product, and the artificial budding defective 1326 aa read-through HIV-1 IIIB Gag–Pol–Nef (GPN) fusion protein as an intracellular product (Gómez et al., 2007b). In particular, the increased magnitude observed in the HIV-1-specific CD8 T cell response appeared to be related with the ISGylation activity. To extend our previous results, here we have defined in mice the immunomodulatory role of either the wild-type ISG15GG or mutated ISG15AA when delivered by the attenuated MVA vector in heterologous combination with the recombinant MVA-B. Our results demonstrate that expression of ISG15AA from MVA-Δ3-ISG15AA significantly enhances the magnitude of HIV-1-specific CD8 T cells, indicating that ISG15 expressed by viral vectors, such as poxviruses, should be taken into consideration as a novel component to be incorporated in prime/boost immunization regimens against HIV-1.

## Materials and methods

### Cell types

BHK-21 cells (a baby hamster kidney cell line; ATCC Cat. No. CCL-10, Manassas, VA, USA), A549 cells (an adenocarcinomic human alveolar basal epithelial cells; ATCC Cat. No. CCL-185), iBMDM (immortalized murine Bone Marrow-Derived Macrophages, generated by Jesús Balsinde and María Ángeles Balboa, Insituto de Génetica y Biología Molecular (IBGM-CSIC Valladolid, Spain), following the protocol previously described (De Nardo et al., 2018) were grown in 1000 mg/L glucose Dulbecco’s Modified Eagle’s Medium (DMEM) (Invitrogen Gibco, New York, USA) supplemented with 10 mM HEPES (Invitrogen Gibco), penicillin (100 U/ml; Sigma-Aldrich, St. Louis, MO, USA), streptomycin (100 μg/ml; Sigma-Aldrich), L-glutamine (2 mM; Sigma-Aldrich), non-essential amino acids (0.1 mM; Sigma-Aldrich), amphotericin B (Fungizone, 0.5 μg/ml; Invitrogen Gibco) and 10% heat-inactivated fetal bovine serum (FBS) (Invitrogen Gibco). Cell cultures were maintained at 37°C in a humidified incubator containing 5% CO_2_.

### Viruses

The poxvirus strains used in this study included the attenuated wild-type Modified Vaccinia virus Ankara (MVA-WT) obtained from the Chorioallantois Vaccinia virus Ankara (CVA) strain after 586 serial passages in chicken embryo fibroblast (CEF) cells (kindly provided by G. Sutter, Ludwig-Maximilians-University of Munich, Munich, Germany), the recombinant virus MVA-B that simultaneously expresses the full-length HIV-1 Gag–Pol–Nef (GPN) fusion protein as an intracellular product and the monomeric gp120 as a cell-released product of clade B from the viral thymidine kinase (TK) locus (*J2R* gene) (Gómez et al., 2007b) and the recombinant virus MVA-Δ3-GFP (kindly provided by Juan García-Arriaza, CNB-CSIC, Madrid, Spain) containing deletions in vaccinia virus (VACV) immunomodulatory genes *C6L*, *K7R* and *A46R* and with the green fluorescent protein (GFP) gene inserted into the TK locus of the MVA-WT genome under the transcriptional control of the VACV synthetic early/late (pE/L) promoter (García-Arriaza et al., 2014a). MVA-Δ3-GFP was used as the parental virus for the generation of MVA-Δ3-ISG15AA and MVA-Δ3-ISG15GG recombinant viruses. All virus infections were performed with DMEM-2% FBS.

### DNA vectors

DNA vectors used in this work included the plasmids pCMV-φ and pCMV-gp120 Bx08 (encoding the HIV-1 clade B Bx08 gp120 protein) previously described (Gómez et al., 2007b). Plasmids were purified using the EndoFree Plasmid Mega kit (Qiagen, Hilden, Germany) and diluted for injection in endotoxin-free phosphate-buffered saline (PBS).

### Construction of the plasmid transfer vectors pCyA20-ISG15GG and pCyA20-ISG15AA

For the generation of the plasmid transfer vectors pCyA20-ISG15GG and pCyA20-ISG15AA, amino-terminal V5-tagged murine ISG15 mature forms of 15 KDa that exposes a carboxy-terminal LRLRGG (ISG15GG) or LRLRAA (ISG15AA) sequences were cloned into the pCyA20 vector previously described (Gómez et al., 2013) using the Gibson Assembly^®^ Kit (New England BioLabs, Ipswich, MA, USA) according to manufacturer’s instructions. The correct generation of both plasmids was confirmed by DNA sequence analysis. The direct the insertion of ISG15AA or ISG15GG genes into the viral genome (TK locus) of MVA-Δ3-GFP by homologous recombination.

### Generation of MVA-Δ3-ISG15GG and MVA-Δ3-ISG15AA recombinant viruses

For the construction of MVA-Δ3-ISG15GG and MVA-Δ3-ISG15AA viruses, 3x10^6^ BHK-21 cells were infected with parental virus MVA-Δ3-GFP at a 0.02 pfu/cell and transfected 1 h later with 10 μg of plasmids pCyA20-ISG15GG or pCyA20-ISG15AA, using Lipofectamine 2000 reagent (Invitrogen, Carlsbad, CA, USA) according to manufacturer’s recommendations. At 72 hours post-infection (hpi), cells were harvested, lysed by freeze-thaw cycling, sonicated and used for recombinant virus screening. Recombinant MVAs containing ISG15GG or ISG15AA genes and transiently coexpressing the β-Gal marker gene were selected by three consecutive rounds of plaque purification steps in BHK-21 cells stained with 5-bromo-4-chloro-3-indolyl-β-D-galactopyranoside (X-Gal, 1.2 mg/ml; Sigma-Aldrich). In the last 3 purification steps, MVA-based recombinant viruses containing ISG15GG/AA genes and having excised the β-Gal marker gene were identified as non-stained viral foci in BHK-21 cells in the presence of X-Gal substrate and isolated. After these six rounds of plaque purification in the presence of X-Gal substrate, a final plaque was picked up and used to infect BHK-21 cells grown in a 100 mm culture plate. The infected cells were collected and used to produce a crude viral preparation in BHK-21 cells. This viral stock was used to further expand the viruses in BHK-21 cells, followed by virus purification through one 45% (w/v) sucrose cushion and a 20-45% (w/v) sucrose gradient to obtain purified stocks for *in vivo* assays. Viral titers were determined by immunostaining plaque assay in BHK-21 cells as previously reported (Ramírez et al., 2000) using rabbit polyclonal anti-VACV strain Western Reserve (WR) antibody (CNB-CSIC), followed by goat anti-rabbit-horseradish peroxidase (HRP) antibody (Sigma-Aldrich). Immunostained palques were reveled with TrueBlue™ Peroxidase Substrate (SeraCare, Milford, MA, USA). The corresponding virus stocks were confirmed to be free of bacterial, fungal and mycoplasma contaminations.

### PCR analysis of MVA-Δ3-ISG15GG and MVA-Δ3-ISG15AA recombinant viruses

To confirm the identity and purity of both MVA-based recombinant viruses, viral DNA was extracted from BHK-21 cells that were infected at of 5 pfu/cell with MVA-WT, MVA-Δ3-GFP, MVA-Δ3-ISG15GG or MVA-Δ-ISG15AA for 24 h. The cell membranes were disrupted by proteinase K treatment (0.2 mg/ml proteinase K in 50 mM Tris-HCl pH 8, 100 mM EDTA pH 8, 100 mM NaCl, 1% SDS; 1 h, 55°C), followed by incubation with RNase A (80 µg/mL). Viral DNA was precipitated using 2-propanol. Primers TK-L: 5’-TGATTAGTTTGATGCGATTC-3’ and TK-R: 5’-CTGCCGTATCAAGGACA-3’ spanning TK flanking regions were used for PCR analysis of TK locus. The amplification reactions were carried out with Phusion High-Fidelity DNA polymerase (New England BioLabs) according to manufacturer´s recommendations.

### Genetic stability and grow profile of MVA-Δ3-ISG15GG and MVA-Δ3-ISG15AA recombinant viruses

The stability of ISG15GG and ISG15AA antigens expressed by the corresponding MVA vectors was analyzed by serial passages in BHK-21 cells. For this, monolayers of BHK-21 cells were infected with MVA-Δ3-ISG15GG or MVA-Δ3-ISG15AA viruses at 0.05 pfu/cell, and after 48–72 hpi cells were collected by scrapping, freeze-thawed three times and cellular extract was used to infect a monolayer of BHK-21 cells for a new round of infection. This process was serially repeated 9 times. The correct expression of ISG15GG and ISG15AA antigens in passages P1 to P9 was analyzed by western-blot (in the following section). To determine the growth profile of both MVA-based recombinant viruses, monolayers of permissive (BHK-21 cells) or non-permissive (A549 cells) cell lines were infected at 0.1 pfu/cell with MVA-Δ3-GFP, MVA-Δ3-ISG15GG or MVA-Δ3-ISG15AA viruses. At different times post-infection (0, 24 and 48 h), cells were harvested by scraping, centrifuged for 5 min at 660 g, supernatant removed, 0.1 mL of complete DMEM added to the cellular pellet, freeze-thawed three times, and briefly sonicated. Viral titers in cell lysates were determined by immunostaining plaque assay. Additionally to analyze ISG15 proteins expressed from MVA-Δ3-ISG15GG or MVA-Δ3-ISG15AA recombinant viruses, monolayers of iBMDM were mock-infected or infected with MVA-Δ3-GFP, MVA-Δ3-ISG15GG or MVA-Δ3-ISG15AA at 5 pfu/cell for 6 and 16 hours. Pellet and supernatant samples were collected separately and anlayzed by western-blotting.

### Analysis of ISG15 protein expression by western-blotting

To analyze ISG15 expression, cellular lysates and supernatants collected were respectively resuspended in Laemmli 1X (2% SDS, 10% Glycerol, 60 mM Tris-Cl pH 6.8, 0.01% Bromophenol Blue and 20 mM of Dithiothreitol) or 5X and fractionated by 12% Sodium Dodecyl Sulfate Polyacrylamide Gel Electrophoresis (SDS-PAGE). Western-blot analysis was performed using the rabbit monoclonal anti-V5 Tag antibody (Cell Signaling, Danvers, MA, USA) to evaluate viral ISG15 expression or ISGylation. For the *in vivo* experiment, collected lungs were suspended at 1g/ml in Laemmli 1X and mechanically processed using a bead beating tissue homogenizer using the FastPrep-24 (Grupo taper, Madrid, Spain) at 45m/s combined with ceramic beads. The homogenized samples were centrifugated and a western-blotting analysis was performed using a monoclonal Armenian hamster ISG15-specific antibody (Invitrogen).

The mouse monoclonal anti-β-actin antibody (Cell Signaling) was used as loading control and a specific antibody against VACV E3 (kindly provide by B. Jacobs, Arizona State University, Tempe, AZ, USA) protein as infection control. Horseradish peroxidase (HRP) -conjugated goat anti-rabbit, anti-mouse or anti Armenian hamster (Sigma-Aldrich) antibodies were used as secondary antibodies. Immune complexes were detected by enhanced chemiluminescence system using Clarity Western ECL substrate (Bio-Rad, Hercules, CA, USA) and imaged via the ChemiDoc system (Bio-Rad).

### Reverse trascription-quantitative real-time polymerase chain reaction (RT-qPCR)

Pellets were collected from BMDM cells that had been infected with MVA-Δ3-GFP, MVA-Δ3-ISG15GG, or MVA-Δ3-ISG15AA viruses at 5 pfu/cell for 6 and 16 hours. The pellets were then suspended in NucleoZOL reagent (MACHEREY-NAGEL, Düren, Germany) following the manufacturer’s instructions. Additionally, pellet samples were collected from iBMDM cells that had been infected with MVA-B, either alone or in combination with MVA-Δ3-ISG15GG or MVA-Δ3-ISG15AA at 1 pfu/cell, at 24 hours post-infection using the same NucleoZOL suspension protocol to obtain RNA. Subsequently, cDNA was obtained from 1 µg of RNA samples using High-Capacity cDNA Reverse Transcription Kit with protease inhibitor (Thermo Fisher Scientific, Waltham, MA, USA). The cDNA obtained was diluted 1:100 in endonuclease free water and the expression of *Isg15*, *Ifn-I*, *Il-6*, *Pkr* and *Tnf-α* genes was analyzed with qPCRBIO SyGreen Mix Hi-ROX (PCR Biosystems, Oxford, UK). RT-qPCR was performed using StepOnePlus System™ (Thermo Fisher Scientific) following the manufacture protocol. Expression levels of *Isg15*, *Ifn-I*, *Il-6*, *Pkr* and *Tnf-α* were analyzed using specific oligonucleotides (**Table 1**). The hypoxanthine-guanine phosphoribosyltransferase (*Hprt*) gene was used as a reference housekeeping gene since its expression remains constant in both infected and non-infected cells. All samples were tested in technical duplicate and at least four different biological replicates were analyzed *in vitro* samples or pooles *in vivo* samples case.

**Table 1:** Murine primers used the this work.

Gene	Forword (5’-3’)	Reverse (5’-3’)
***Hprt***	GATTAGCGATGATGAACCAGGTT	CCTCCCATCTCCTTCTTCATGACA
***Ifn-β***	TGACTGTGAGAGCAAGCAGC	CCCCAGCATCTTCACCTTTA
***Il-6***	GTATGAACAACGATGATGCACTTG	ATGGTACTCCAGAAGACCAGAGGA
***Isg15***	GACGGTCTTACCCTTTCCAGT	CCTTTCGTTCCTCACCAGGAT
***Pkr***	GGAAAATCCCGAACAAGGAG	CCCAAAGCAAAGATGTCCAC
***Tnf-α***	ATGAGCACAGAAAGCATGA	AGTGACAGAAGAGCGTGGT

Target gene (column A); Forward primer sequence (5’-3’) (column B); Reverse primer sequence (5’-3’) (column C).

### IFN-α ELISA assay

IFN-α concentration was quantified in fresh supernatants from iBMDM mock- or MVA-Δ3-GFP-infected at 5 pfu/cell and collected at 6 and 16 hpi. Quantification was performed using a commercial IFN alpha Mouse ELISA Kit (Invitrogen) following the manufacturer´s instructions.

### Viral inoculation of mice and sample collection

Female C57BL/6 mice (6-8 week-old) purchased from Charles River (Hopkinton, MA, USA) were used to evaluate the local effect of ISG15GG or ISG15AA in lungs. For this, a total of 20 mice were divided in the following four groups: G1: PBS (negative control); G2: MVA-Δ3-GFP; G3: MVA-Δ3-ISG15GG and G4: MVA-Δ3-ISG15AA (n=5 per group). Mice were anesthetized with isoflurane and infected intranasally with 1 x 10^6^ pfu of the different MVAs or with PBS alone. After 48 h mice were sacrificed and lungs were harvested during necropsy. Lungs were suspended in NucleoZOL reagent (MACHEREY-NAGEL) for RT-qPCR, or immersion-fixed in 4% formalin for 48 hours. After fixation period, samples were routinely processed and embedded in paraffin blocks that were then sectioned at 4 µm thickness on a microtome, mounted onto glass slides and routinely stained with haematoxylin and eosin (H&E) (Histology Service, CNB-CSIC). Images were acquired using a Nikon eclipse TS100 microscope.

### Peptides

The HIV-1 clade B consensus peptide pools used in this work include Env-1 (63 peptides), Env-2 (61 peptides), Gag-1 (55 peptides), Gag-2 (50 peptides), GPN-1 (53 peptides), GPN-2 (57 peptides), GPN-3 (56 peptides) and GPN-4 (55 peptides). They were provided by the National Institutes of Health (NIH) AIDS Research and Reference Reagent Program (Bethesda, MD, USA) and covered the HIV-1 clade B Env, Gag, Pol and Nef proteins included in the HIV-1 antigens expressed from DNA-B and MVA-B vectors as consecutive 15-mers overlapping by 11 amino acids. To analyze the HIV-1-specific cellular immune responses, we combined the above peptide pools as follows: Env pool (Env-1+Env-2), Gag pool (Gag-1 + Gag-2) and GPN pool (GPN-1 + GPN-2 + GPN-3 + GPN-4). VACV E3_140-148_ peptide (sequence: VGPSNSPTF; CNB-CSIC Proteomics Service, Madrid, Spain), previously described as an immunodominant epitope in BALB/c mice (Tscharke et al., 2006), was used to determine VACV-specific CD8 T cell responses.

### Mouse immunizations

Female BALB/c mice (6-8 week-old) were purchased from Envigo Laboratories (Indianapolis, IN, USA) and stored in the animal facility of the CNB (Madrid, Spain). To characterize the immunostimulatory effect of ISG15GG or ISG15AA expressed from MVA vector on the HIV-1-specific acute immune response, four groups of BALB/c mice (n=4) were immunized with 50 µg of pCMV-gp120-B (shortly DNA-B; groups 1-3) or pCMV-φ (shortly DNA-φ; group 4) by bilateral intramuscular (i.m.) route. Four weeks later, mice were immunized by intraperitoneal (i.p.) route with 1×10^7^ total pfu of the following MVA-based vector combinations (5×10^6^ pfu of each virus): G1: MVA-WT + MVA-Δ3-GFP; G2: MVA-B + MVA-Δ3-GFP; G3: MVA-B + MVA-Δ3-ISG15GG and G4: MVA-B + MVA-Δ3-ISG15AA. At 10 days post-boost, animals were sacrificed and spleens were processed for Intracellular Cytokine Staining (ICS) assay to determine cellular HIV-1-specific acute immune response.

### Analysis of the HIV-1-specific T cell immune response by ICS assay

To analyze the magnitude and phenotype of the HIV-1- or VACV-specific T cell immune responses, 2×10^6^ splenocytes (erythrocyte-depleted) seeded on 96-well plates were stimulated *ex vivo* for 6 h in Roswell Park Memorial Institute-1640 medium (RPMI-1640; Sigma-Aldrich) supplemented with 2 mM L-glutamine, 100 U/mL penicillin/100 μg/mL streptomycin, 10 mM Hepes, 0.01 mM β-mercaptoethanol and 10% fetal calf serum (FCS) (Sigma-Aldrich), 1 μL/mL Golgiplug (BD Biosciences, San Jose, CA, USA), monensin 1X (Invitrogen), anti-CD107a-FITC (BD Biosciences) and 5 µg/mL of the HIV-1 clade B consensus peptide pools or 10 µg/mL of VACV E3 peptide. Non-stimulated samples (RPMI) were used as control. After stimulation, splenocytes from immunized mice were washed, stained for surface markers, fixed/permeabilized (Cytofix/Cytoperm kit; BD Biosciences) and stained intracellularly. For the analysis of CD4 and CD8 T cell immune responses, the following fluorochrome-conjugated antibodies were used: CD107a-FITC, IFN-γ-PE-Cy7, IL-2-APC and TNF-α-PE for functional analyses and CD3-PE-CF594, CD4-APC-Cy7 and CD8-V500 for phenotypic analyses. All antibodies were from BD Biosciences. Dead cells were excluded using the LIVE/DEAD Fixable Violet Dead Cell Stain Kit (Invitrogen). Cells were acquired in a GALLIOS flow cytometer (Beckman Coulter, Brea, CA, USA) and data analyses were carried out using FlowJo software (Version 10.4.2; Tree Star, Ashland, OR, USA). Lymphocyte-gated events ranged between 10^5^ and 5×10^5^. After gating, boolean combinations of single functional gates were generated to quantify the frequency of each response based on all the possible combinations of cytokine expression or differentiation markers. Background responses in the unstimulated controls (RPMI) were subtracted from those obtained in stimulated samples for each specific functional combination.

### Ethics statement

Animal experimental protocols were approved by the Ethical Committee of Animal Experimentation (CEEA) of Centro Nacional de Biotecnología (CNB-CSIC, Madrid, Spain) and of the Universidad Autónoma de Madrid (UAM) according to Spanish National Royal Decree RD 53/2013, Spanish National Law 32/2007 on animal welfare, exploitation, transport and sacrifice and International EU Guidelines 2010/63/UE on protection of animals used for experimentation and other scientific purposes (permit number PROEX 281/16 and PROEX 184.3/22).

### Data analysis and statistics

The statistical analyses of viral growth kinetics, RT-qPCR and ELISA data were carried out with GraphPad 9 using a statistical one way or two–way analysis of variance (ANOVA) with a Tukey *post hoc* test correction for multiple comparisons when applied. For the analysis of ICS data, a statistical approach that adjusts the values for the non-stimulated controls (RPMI) and calculates the confidence intervals and p values was used (Nájera et al., 2010). Only antigen responses significantly higher than the corresponding RPMI samples are represented and background-subtracted. Significant differences are described as follows: *p ≤ 0.05; **p ≤ 0.005; ***p ≤ 0.001, ****p ≤ 0.0001.

## Results

### *In vitro* characterization of MVA-Δ3-ISG15GG and MVA-Δ3-ISG15AA recombinant viruses

**Figure 1:**
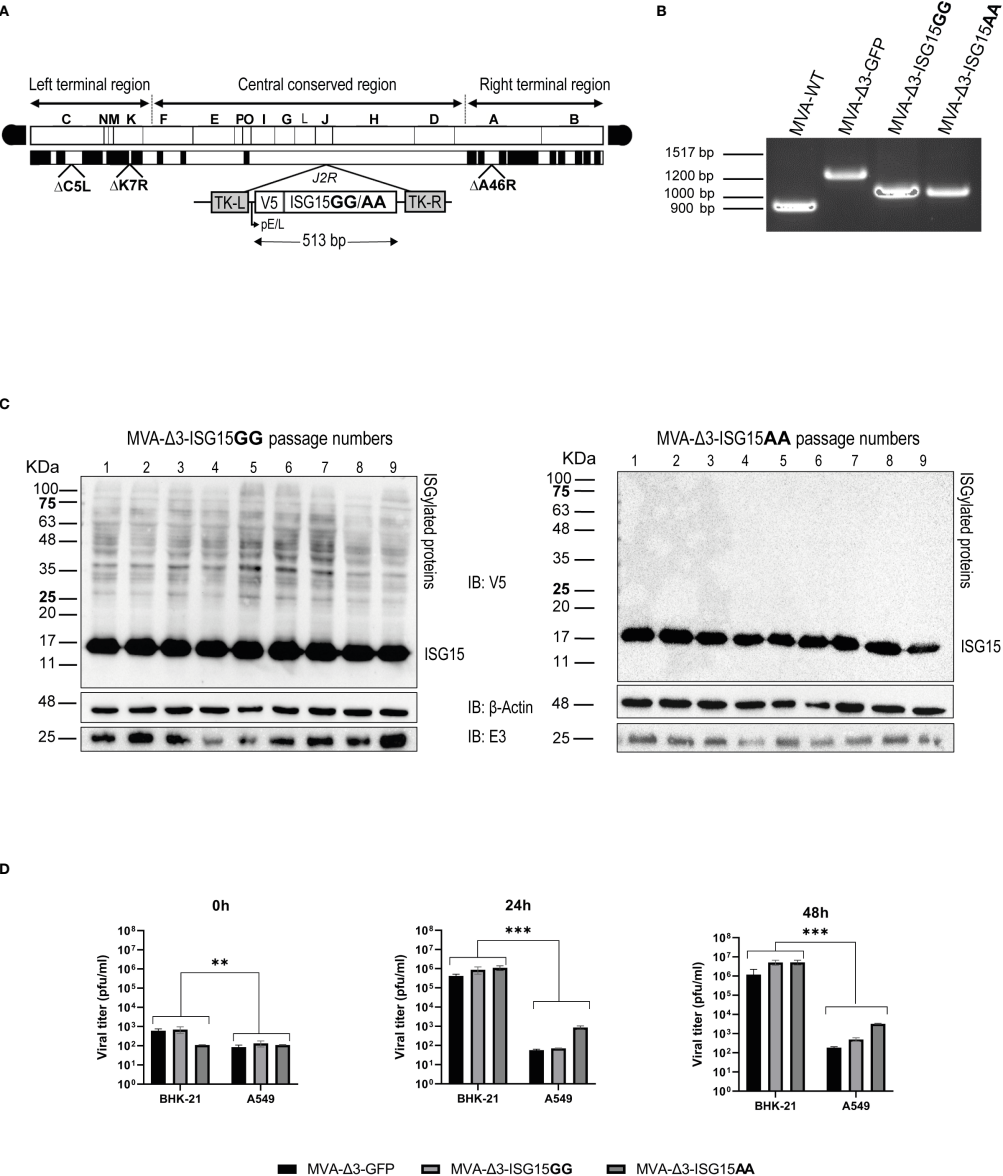
Generation and *in vitro* characterization of MVA-Δ3-ISG15GG and MVA-Δ3-ISG15AA recombinant viruses. **(A)** Scheme of the genome of both ISG15-expressing MVA viruses. ISG15 genes were inserted into the *TK* locus of parental MVA-Δ3-GFP virus. The locations of *C6L, K7R* and *A46R* genes deleted in MVA-Δ3-GFP-based viruses are indicated. TK-L: TK left flanking region; TK-R: TK right flanking region. **(B)** PCR analysis of the VACV TK locus. Viral DNA was extracted from BHK-21 cells infected with MVA-WT, MVA-Δ3-GFP, MVA-Δ3-ISG15GG or MVA-Δ3-ISG15AA. Primers spanning TK flanking regions were used for the analysis of TK locus by PCR. **(C)** Genetic stability of MVA-Δ3-ISG15GG and MVA-Δ3-ISG15AA recombinant viruses. Western-blotting analysis of successive passages of MVA-Δ3-ISG15GG (left panel) and MVA-Δ3-ISG15AA (right panel) recombinant viruses in BHK-21 cells using an antibody against V5 Tag. β-actin and E3 were used as loading and VACV-infection controls, respectively. The positions of free-ISG15 and ISGylated proteins are indicated on the right. **(D)** Virus growth kinetics. Monolayers of permissive (BHK-21) or non-permissive (A549) cells were infected at 0.01 pfu/cell with the parental MVA-Δ3-GFP or with MVA-Δ3-ISG15GG or MVA-Δ3-ISG15AA viruses. At 0, 24 and 48hpi, infected cells were harvested and virus titers in cell lysates were quantified by plaque immunostaining assay. Mean ± SD of two independent experiments is shown. **p ≤ 0.005; ***p ≤ 0.001.

Considering the adjuvant capacity of ISG15, we aimed to express this molecule from an immunomodulatory vector such as the poxvirus MVA strain. To define whether we could enhance its adjuvant ability and to test the role of ISGylation on it, we generated two MVA-Δ3-GFP-based recombinant viruses expressing V5-tagged ISG15GG (wild-type form) or ISG15AA (a mutant form, unable to perform ISGylation) proteins, as described in Materials and Methods section. The scheme of the DNA genome of both recombinant viruses is depicted in **Figure 1A**. For ISG15 gene insertion, we used as parental virus an MVA-Δ3-GFP vector lacking three VACV immunomodulatory genes with improved efficacy as vaccine vector (García-Arriaza et al., 2011; García-Arriaza et al., 2013).

The insertion of *ISG15* gene into the *J2R* locus of MVA-Δ3-GFP genome, as well as the purity of both recombinant viruses, was determined by PCR with primers spanning the TK flanking regions. As shown in **Figure 1B**, in MVA-WT and in parental MVA-Δ3-GFP, 873 bp- and 1226 bp-products were observed, respectively. However, in MVA-Δ3-ISG15GG and MVA-Δ3-ISG15AA samples a unique 1025-bp product was obtained, indicating that ISG15 protein were successfully inserted into the viral TK locus and no parental contamination was present in MVA-Δ3-ISG15GG and MVA-Δ3-ISG15AA viral preparations. These results were also confirmed by DNA sequence analysis.

To assess the stability of the ISG15 proteins expressed by MVA-Δ3-ISG15GG and MVA-Δ3-ISG15AA viruses, we performed serial passages on a permissive cell line (BHK-21) and analyzed ISG15 expression by western-blotting using an anti-V5 tag antibody. As shown in **Figure 1C**, both recombinant viruses efficiently expressed ISG15 protein up to 9 consecutive passages, indicating stable insertion of the heterologous *ISG15* gene into the MVA genome. The correct expression of the ISG15 protein was also confirmed by the presence of ISGylation after MVA-Δ3-ISG15GG infection (noted by smear of ISGylated protein) and its absence after infection with the mutated MVA-Δ3-ISG15AA. In addition, we conducted viral growth analyses in permissive (BHK-21) and non-permissive (A549) cell lines to determine whether in cultured cells the *ISG15* gene insertion affected the replication and virus growth of MVA-based recombinant viruses. As observed in **Figure 1D**, in BHK-21 cells, both recombinant viruses showed similar growth kinetics, roughly from 10^3^ pfu/mL (t=0 hpi) to 10^6^ (t=24 hpi), or 10^7^ pfu/mL (t=48 hpi). However, in a human non-permissive cell line (A549), the viral titers barely increase with time, approximately from 10^2^ pfu/mL (t=0 hpi)) to 10^2^ (t=24 hpi), 10^2^ pfu/mL (t=48 hpi), indicating that as expected MVA is unable to efficiently replicate in human cell lines (Nájera et al., 2006).

Overall, these results indicated that the insertion of wild-type and mutated ISG15 sequences into the MVA-Δ3-GFP genome did not impair its replication and growth properties under permissive conditions, maintaining its restriction in human cells.

### Effect of ISG15 expression in MVA-Δ3-ISG15-infected macrophages

**Figure 2:**
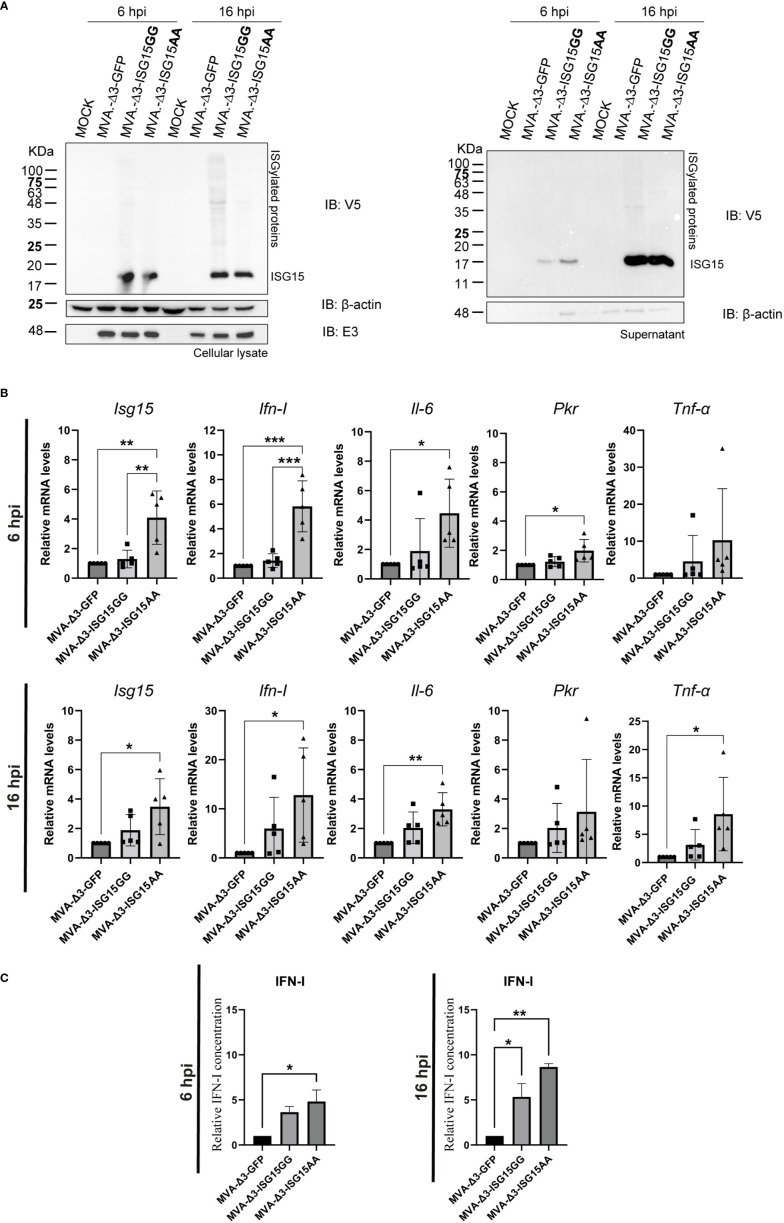
*In vitro* effect of ISG15 expression from MVA-Δ3-ISG15-infected macrophages. **(A)** Time-course expression of ISG15GG and ISG15AA proteins in cellular extracts (left panel) and supernatants (right panel) from iBMDM mock-infected or infected with MVA-Δ3-GFP, MVA-Δ3-ISG15GG or MVA-Δ3-ISG15AA viruses at 5 pfu/cell for 6 and 16 hours. Western-blotting analysis was performed using a mouse monoclonal anti-V5 antibody for the detection of V5-ISG15 proteins. β-actin and E3 were used as loading and VACV-infection controls, respectively. The positions of free-ISG15 and ISGylated proteins are indicated on the right. **(B)** RT-qPCR analysis of the expression of *Isg15, Ifn-I*, *Il-6*, *Pkr* and *Tnf-α* genes in iBMDM mock-infected or infected at 5 pfu/cell with MVA-Δ3-GFP, MVA-Δ3-ISG15GG or MVA-Δ3-ISG15AA at 6 (upper panel) and 16 (bottom panel) hpi. The mRNA expression levels of each gene were analyzed by RT-qPCR and are expressed relative to the endogenous *Hprt* gene, not influenced by infection, and subtracted from the levels obtained in mock-infected cells. Mean ± SD of 5 biological replicates is represented. Each symbol (dot, triangle or cube) represents an independent experiment of the correspondent group. **(C)** Analysis of IFN-I secretion in the supernatants from iBMDM mock- or MVA-Δ3-infected cells at 5 pfu/cell at 6 (left panel) and 16 hpi (right panel). Mean ± SD of 2 technical replicates is represented. *p ≤ 0.05; **p ≤ 0.005; ***p ≤ 0.005.

Macrophages are innate immune cells specialized in the detection, phagocytosis and destruction of pathogens. These cells are involved in antigen presentation to T cells and in the inflammation process by releasing molecules, such as cytokines, that activate other cells and induce reactive oxygen species production (Lendeckel et al., 2022). The characterization of the properties of the MVA-Δ3-based recombinant viruses in a key immune cell line such as macrophages represents the first *in vitro* approach to understand the features of ISG15 when expressed from a viral vector. Hence, we analyzed by western-blotting the V5-tagged ISG15 expression from MVA-Δ3 vectors using a specific antibody against V5 epitope in cellular extracts and supernatants from iBMDM mock-infected or infected with MVA-Δ3-GFP, MVA-Δ3-ISG15GG or MVA-Δ3-ISG15AA at 6 and 16 hpi. As observed in **Figure 2A**, both MVA-Δ3-ISG15GG and MVA-Δ3-ISG15AA viruses express the heterologous ISG15 proteins in infected macrophages. In cellular lysates (**Figure 2A** **, left panel**), ISG15 was detected in cells infected with both viruses at 6 hpi and ISGylation was only observed at 16 hpi in cells infected with MVA-Δ3-ISG15GG. We also analyzed the presence of secreted ISG15 in the supernatants of infected cells. Cell-free ISG15 was detected at 6 hpi in the supernatants of cells infected with both viruses (**Figure 2A** **, right panel**), with levels markedely increased by 16 hpi. These results confirmed the expression of ISG15 proteins in infected macrophages, and indicate that ISG15 proteins produced from MVA vectors are able to be conjugated and secreted.

To better understand whether ISGylation has an impact on the immune response, we analyzed by RT-qPCR the transcription mRNAs levels of *Isg15*, *Ifn-I, Il-6*, *Pkr* and *Tnf-α* murine genes from iBMDM mock-infected or infected with MVA-Δ3-GFP, MVA-Δ3-ISG15GG or MVA-Δ3-ISG15AA at 5 pfu/cell at 6 and 16 hpi (**Figure 2B**). Our results indicate an increase of *Isg15*, *Ifn-I, Il-6* and *Pkr* mRNA levels at 6 hours after MVA-Δ3-ISG15AA infection but not in the MVA-Δ3-ISG15GG-infected cells (**Figure 2B** **, upper panel**). These upregulated genes were maintained at 16 hpi (**Figure 2B** **, bottom panel**).

To evaluate if the increased *Ifn-I* mRNA levels was correlated with increased secretion of the protein, we quantified IFN-I levels in the supernatants from uninfected or infected iBMDM at 6 and 16 hpi by enzyme-linked immunosorbent assay (ELISA). As shown in **Figure 2C**, both MVA recombinants expressing the wild-type (ISG15GG) or the mutated (ISG15AA) ISG15 forms induced the secretion of IFN-I in infected macrophages at 6 and 16 hpi compared with the parental MVA-Δ3-GFP, with the highest levels detected after MVA-Δ3-ISG15AA infection (**Figure 2C** **, right panel**).

### Effect of ISG15 expression in mice after intranasal administration of MVA-Δ3 vectors

**Figure 3:**
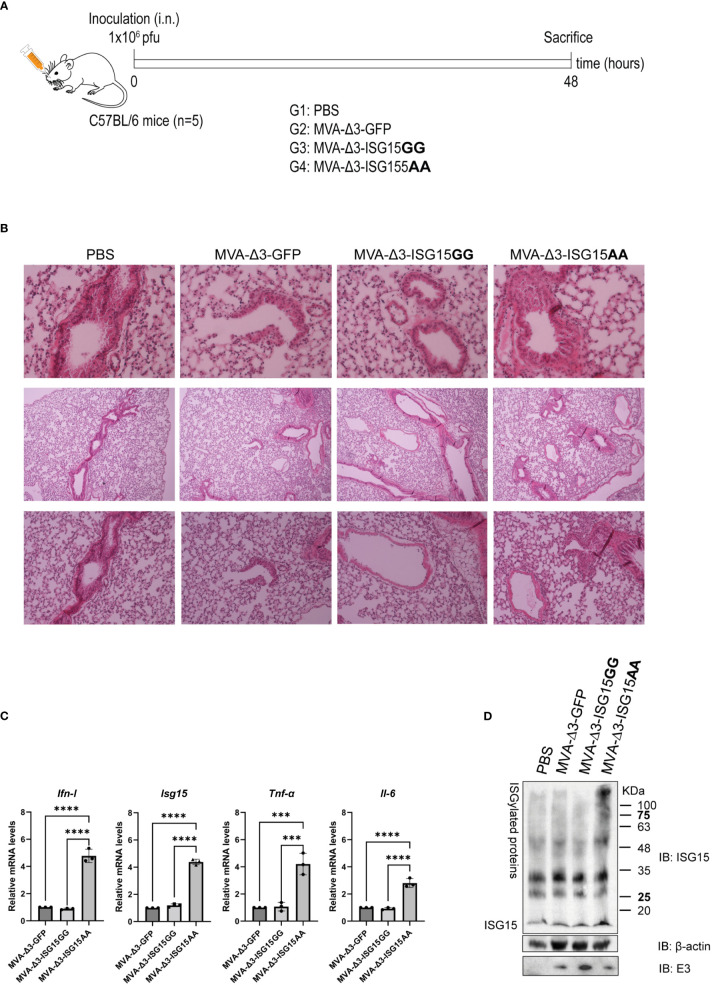
*In vivo* intranasal inoculation of mice with MVA-Δ3-based recombinant viruses expressing ISG15. **(A)** Infection schedule. C57BL/6 mice (n=5) were infected i.n. with 1x10^6^ pfu of MVA-Δ3-GFP, MVA-Δ3-ISG15GG or MVA-Δ3-ISG15AA viruses or PBS-treated as control group. At 48 hpi mice were sacrificed and lungs harvested. **(B)** Representative histological images of H&E stained sections of infected mouse lungs. **(C)** Evaluation of mRNA levels of *Ifn-I, Isg15, Tnf-α*, and *Il-6* genes in lungs (pool) from infected mice by RT-qPCR. The mRNA expression levels of each gene were analyzed by RT-qPCR and are expressed relative to the endogenous *Hprt* gene, not influenced by infection, and subtracted from the levels obtained in PBS-infected mice. Mean ± SD of 3 technical replicates is represented. **(D)** Evaluation of ISGylation in lungs of infected mice. Lung lysates (pool) were fractionated by 12% SDS-PAGE and ISGylation levels were analyzed using a mouse monoclonal anti-ISG15 antibody. Positions of the free-ISG15 form and of the ISGylated proteins are indicated. β-actin and VACV E3 protein were used as loading and infection controls, respectively. ***p ≤0.001, ****p ≤0.0001.

To test whether the *in vitro* production of IFN-I after infection with MVA recombinants had an impact *in vivo*, we decided to characterize the effect of the infection in the murine model. For this, C57BL/6 mice (n=5) were infected intranasally (i.n.) with 1x10^6^ pfu of MVA-Δ3-GFP, MVA-Δ3-ISG15GG or MVA-Δ3-ISG15AA or with PBS as control. After 48 hpi, mice were sacrificed and lungs were fixed to evaluate the lung pathology. This time was selected since expression of foreign antigens from the MVA vector is only transient and up to 48 hpi (Gómez et al., 2007a). The schedule and immunization groups are depicted in **Figure 3A**. After hematoxylin and eosin (H&E) staining, no histological differences in terms of tissue damage, infiltration of lymphocytes or blood vessel inflammation were observed in lungs from animals infected with any of the recombinants compared to PBS-treated mice (**Figure 3B**). Thus, we can conclude that both vectors are safe in the murine model.

With the aim to evaluate the proinflammatory cytokine profile in infected lungs, mRNA levels of *Ifn-I*, *Isg15 Tnf-α* and *Il-6* were analyzed by RT-qPCR. As observed in **Figure 3C**, MVA-Δ3-ISG15AA infection produced a significant increase in mRNA levels of the four genes analyzed, validating the *in vitro* results obtained in macrophages. In addition, we also evaluated the endogenous ISGylation levels by western-blotting in the infected tissue **Figure 3D**. Surprisingly, a marked increase of ISGylation levels was observed in lungs from animals infected with MVA-Δ3-ISG15AA virus, indicating that the endogenous ISGylation was enhanced after the overexpression of the mutant ISG15AA by the MVA vector. These results suggest that the increased *Ifn-I*mRNA levels detected in lungs from animals after MVA-Δ3-ISG15AA infection could exert an effect in the increase of ISGylation levels. Therefore, we can conclude that MVA-Δ3-ISG15AA virus is a strong IFN-I inducer and, consequently, an ISG15 and ISGylation enhancer during *in vivo* infection.

**Figure 4:**
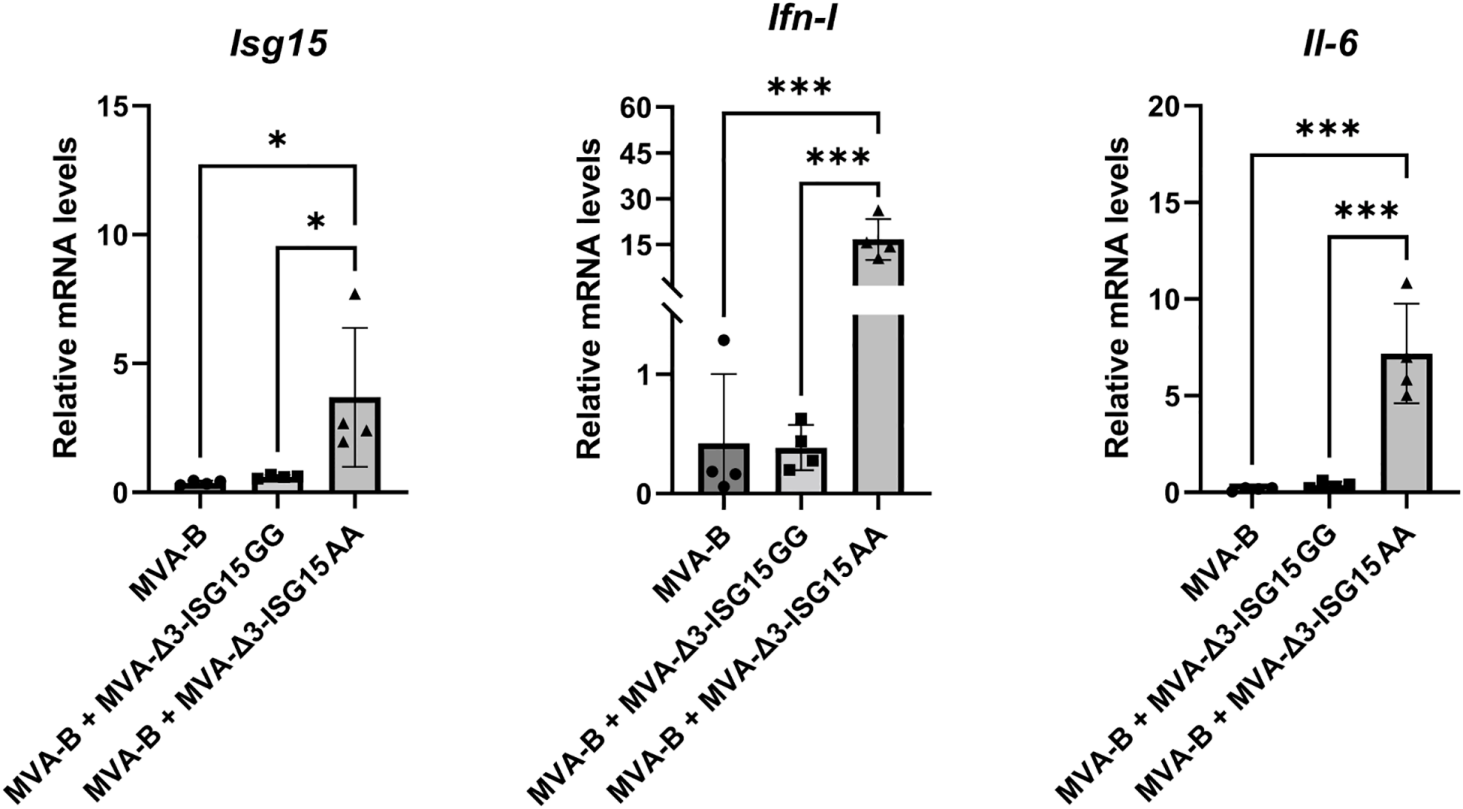
*In vitro* iBMDM co-infection of MVA-B and MVA-Δ3-based recombinant viruses expressing ISG15. mRNA analysis of the expression levels of *Isg15*, *Ifn-I* and *Il-6* genes by RT-qPCR. iBMDM were mock- or MVA-infected as indicated at 1 pfu/cell at 24 hpi mRNA was collected and the expression of *Isg15, Ifn-I* and *Il-6* genes was analyzed by RT-qPCR. The mRNA expression levels of each gene were analyzed in relation to their respective endogenous *Hprt* gene, not influenced by infection, and subtracted from the levels obtained in mock-infected cells. Data shown are relative to MVA-Δ3-GFP infection. Mean ± SD of 4 biological replicates is represented. **p ≤ 0.05; ***p ≤ 0.001.

Since our final goal is to study the immunomodulatory role of both ISG15 recombinat viruses in heterologous combination with the recombinant MVA-B (a vector expressing Env/Gag-Pol-Nef of HIV-1 from clade B), we decided to test *in vitro* whether the expression of HIV-1 antigens from MVA-B in combination with ISG15 expressed from MVA vectors modify the genes analyzed transcription profile induced by MVA-Δ3-ISG15GG or MVA-Δ3-ISG15AA. For this, murine iBMDM cells were infected with MVA-B alone or in combination with MVA-Δ3-ISG15GG or MVA-Δ3-ISG15AA. At 24 hpi, total RNA was extracted to measure mRNA levels of *Isg15*, *Ifn*-*I* and *Il-6* cytokine genes by RT-qPCR. As shown in **Figure 4**, a significant increase in the mRNA expression levels of these cytokines was only detected in iBMDM infected with MVA-Δ3-ISG15AA in combination with MVA-B. These results confirmed our previous findings (**Figure 2B**) and revealed that in macrophages the co-expression of HIV-1 antigens and the mutated ISG15AA form does not modify the transcription profile induced by MVA-Δ3-ISG15AA.

### Co-administration of ISG15AA significantly enhances the magnitude of the HIV-1-specific CD8 T cells

**Figure 5:**
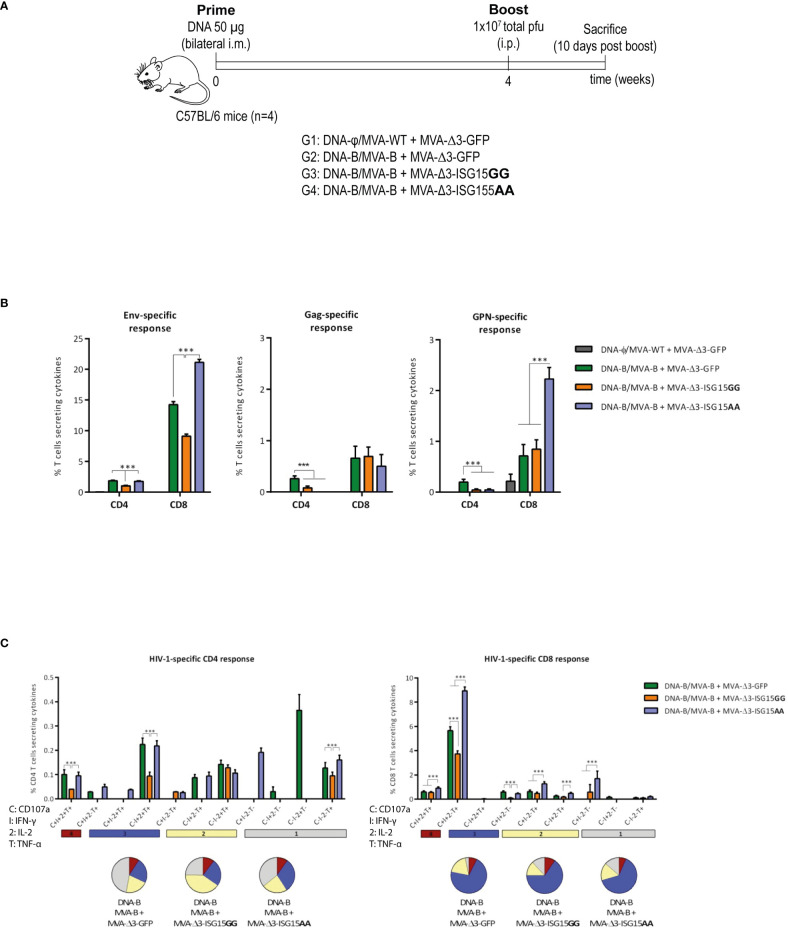
HIV-1-specific T cell acute immune response elicited in mice after DNA-B prime/MVA-B + MVA-Δ3-ISG15 boost. **(A)** Immunization schedule. Four groups of BALB/c mice (n=4) were immunized with 50 µg of DNA-B (groups 1-3) or DNA-φ (group 4) by bilateral i.m. route. Four weeks later, mice were immunized by intraperitoneal i.p. route with 1×10^7^ total pfu of the indicated MVA-based vector combinations. At 10 days post-boost, animals were sacrificed, and spleens were processed for Intracellular Cytokine Staining (ICS) assay to determine cellular HIV-1-specific acute immune response. **(B)** Magnitude of the HIV-1-specific CD4 and CD8 T cell responses measured at 10 days post-boost by ICS assay following *ex vivo* stimulation of splenocytes from immunized mice with the HIV-1 clade B consensus peptide pools. The total value in each group represents the sum of the percentages of CD4 or CD8 T cells secreting IL-2 and/or IFN-γ and/or TNF-α against HIV-1 Env (left panel), Gag (middle panel) or GPN (right panel) clade B consensus peptide pools. All data is background-subtracted. 95% CI is represented. **(C)** Polyfunctional profile of the total HIV-1-specific CD4 (left panel) or CD8 (right panel) T cells in the different immunization groups. The positive combinations of the responses are indicated on the x axis, while the percentages of the functionally different cell populations within the total CD4 or CD8 T cells are represented on the y axis. Specific responses are grouped and color-coded based on the number of functions. C, CD107a; I, IFN-γ; 2, IL-2; T, TNF-α. ***p ≤ 0.001.

The efficacy of MVA-B as a vaccine vector has been previously demonstrated in preclinical and clinical trials (Gómez et al., 2015). In particular, the heterologous combination of DNA encoding HIV-1 gp120 protein (DNA-gp120) as a prime, followed by MVA-B vector as a boost, is an efficient protocol to induce HIV-1-specific B and T cell immune responses in mice (García-Arriaza et al., 2010; García-Arriaza et al., 2011; García-Arriaza et al., 2014b; Pérez et al., 2020). Thus, we decided to use this protocol to explore the immunostimulatory effect of ISG15GG or ISG15AA expressed from MVA vector on the acute phase of the immune response. To this end, four groups of BALB/c mice (n=4) were immunized following the schedule shown in **Figure 5A**. At 10 days post-boost, animals were sacrificed to evaluate in spleen the HIV-1- or MVA vector-specific T cell acute immune responses.

#### HIV-1-specific acute T cell responses

The HIV-1-specific T cell immune responses were evaluated in immunized animals by polychromatic ICS assay after the *ex vivo* stimulation of splenocytes with a pool of overlapping peptides that spanned the HIV-1 Env, Gag, Pol and Nef proteins. In this way, we measured the impact of ISG15 from the values of Env-specific T cell response induced after two doses of gp120, one delivered from the DNA-gp120 vector at priming and the other from the MVA-Env/Gag-Pol-Nef vector after the boost. The magnitude of the HIV-1-specific CD4 and CD8 T cell immune responses was determined as the percentage of T cells within each subset that produced IFN-γ and/or IL-2 and/or TNF-α. The quality of the HIV-1-specific T cells was characterized by the pattern of cytokine production and their cytotoxic potential.

As shown in **Figure 5B**, the HIV-1-specific immune response elicited by the different heterologous combinations of DNA-B prime/MVA-B boost, with or without MVA-Δ3-ISG15 boost, was mainly mediated by CD8 T cells and directed mostly against Env, followed to a lesser extent by GPN and Gag HIV-1 antigens. The co-administration of the mutant ISG15 unable to perform ISGylation (MVA-Δ3-ISG15AA) with MVA-B in the boost, induced the highest HIV-1 Env- and GPN-specific CD8 T cell responses, while for HIV-1 Gag-specific CD8 T cells no differences were detected between the different immunization protocols. In the case of HIV-1-specific CD4 T cells, the levels were low with no significant differences between parental and mutated ISG15, and with the combination of DNA-B/MVA-B + MVA-Δ3-GFP inducing the highest HIV-1-specific CD4 T cells.

We also determined the quality of the HIV-1-specific T cell responses by the pattern of cytokine secretion or degranulation marker CD107a expression on the surface of activated cells. On the basis of the analysis of IFN-γ, IL-2 and TNF-α production or CD107a secretion, eleven (for CD4 T cells) or nine (for CD8 T cells) different HIV-1-specific T cell populations were identified (**Figure 5C**). The percentages of cells producing cytokines or expressing CD107a obtained in the DNA-φ/MVA-WT + MVA-Δ3-GFP control populations were subtracted in the rest of immunization groups in order to remove the non-specific responses detected as background. Vaccine-induced CD8 T cell responses were highly polyfunctional in all immunization groups, with more than 85% of CD8 T cells exhibiting two or three functions. CD8 T cells expressing simultaneously CD107a + IFN-γ + TNF-α were the most representative population, although the absolute frequency of this population was significantly higher in MVA-Δ3-ISG15AA group (**Figure 5C** **, right panel**). Vaccine-induced CD4 T cell responses were less polyfunctional in all immunization groups, with more than 50% of CD4 T cells exhibiting two or three functions. Both MVA-Δ3-ISG15GG and MVA-Δ3-ISG15AA increased the polyfunctionality of the CD4 T cells compared to control group MVA-Δ3-GFP. CD4 T cells expressing simultaneously IFN-γ + IL-2 + TNF-α followed by CD107a + IFN-γ + IL-2 + TNF-α and TNF-α were the most representative populations in the different immunization groups, although the absolute frequencies of these populations were significantly higher in MVA-Δ3-GFP and MVA-Δ3-ISG15AA groups (**Figure 5C** **, left panel**).

#### MVA-specific acute T cell responses

**Figure 6:**
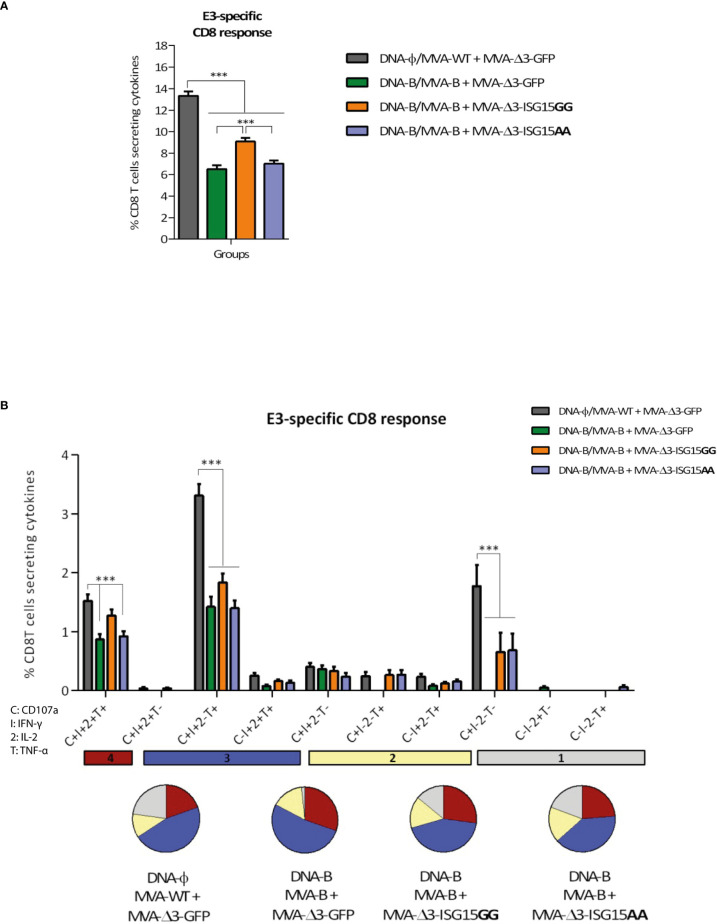
VACV E3-specific CD8 T cell acute immune response elicited in mice after DNA-B prime/MVA-B + MVA-ISG15 boost. **(A)** Magnitude of the VACV E3-specific CD8 T cell responses measured at 10 days post-boost by ICS assay following *ex vivo* stimulation of splenocytes from immunized mice with the VACV E3 peptide. The total value in each group represents the percentage of CD8 T cells secreting IL-2 and/or IFN-γ and/or TNF-α against VACV E3 peptide. All data are background-subtracted. 95% CI is represented. **(B)** Polyfunctional profile of the VACV E3-specific CD8 T cell response in the different immunization groups at 10 days post-boost. The ten positive combinations of the responses are indicated on the x axis, while the percentages of the functionally different cell populations within the total CD8 T cells are represented on the y axis. Specific responses are grouped and color-coded based on the number of functions. C, CD107a; I, IFN-γ; 2, IL-2; T, TNF-α. ***p ≤ 0.001.

Since the MVA vector is used as a boost in all immunization groups, we next decided to evaluate whether ISG15 have any impact on the activation of vector-specific CD8 T cells. For this, MVA-specific responses were measured by polychromatic ICS assay following the protocol described above but using the CD8 T cell-specific peptide from the E3 protein of MVA for the *ex vivo* stimulation of the splenocytes from immunized mice. As shown in **Figure 6A**, the highest E3-specific CD8 T cell immune response was detected in the control group DNA-φ/MVA-WT + MVA-Δ3-GFP, indicating that the HIV-1 antigens act as competitors for the immunodominance of MVA antigens, as we have previously reported (Gómez et al., 2020). Among the other three immunization groups, the combination MVA-B + MVA-Δ3-ISG15GG elicited the highest E3-specifc CD8 T cell response.

The anti-MVA CD8 T cell responses were highly polyfunctional in all groups with more than 75% of the CD8 T cells exhibiting two or more functions (**Figure 6B**). Both MVA-ISG15GG and MVA-ISG15AA decreased the polyfunctionality of the CD8 T cells compared to control group MVA-Δ3-GFP. CD8 T cells expressing simultaneously CD107a + IFN-γ + TNF-α followed by CD107a + IFN-γ + IL-2 + TNF-α and CD107a + IFN-γ were the most prevalent populations in the different immunization groups, although the absolute frequencies of these populations were significantly higher in MVA-Δ3-GFP group.

## Discussion

Current pandemics and the fear of future pandemics highlighted the concept of prevention, emphasizing how the development and the use of effective vaccines can protect people from new outbreaks of viral infections. Among the current pandemics, the HIV/AIDS remains a major global public health challenge to be addressed. The United Nations Programme on HIV/AIDS^2^ and the World Health Organization^3^ reported that 36.3 million people have died from AIDS-related illnesses since the start of the pandemic. Although there is currently no cure for HIV-1 infection, 38.4 million people were living in 2021 with this chronic disease under control thanks to proper diagnosis and treatment with retroviral drugs. Sadly, 650000 people died worldwide from HIV-related illnesses^3^, thus prevention is a key factor in avoiding further spread. Unfortunately, despite enormous efforts to develop a prophylactic HIV vaccine, none of the candidates assessed in phase IIb/III clinical trials have been able to improve the modest 31.2% efficacy against HIV-1 infection demonstrated during the RV144 trial conducted in Thailand (Rerks-Ngarm et al., 2009). Therefore, there is an urgent need to develop optimized immunogens, immunization protocols and adjuvants that can improve these results. From this need arises the idea to investigate in depth the immunomodulatory molecule ISG15, an Interferon-induced protein (Farrell et al., 1979; Haas et al., 1987) that acts as an alarmin by enhancing specific immune responses against HIV-1 (Gómez et al., 2020). ISG15 is a ubiquitin-like protein, present intracellularly conjugated covalently to newly synthesized proteins (ISGylation) or as free form that can be secreted. ISG15 is involved in a plethora of biological pathways (Han et al., 2018; Sandy et al., 2020; Tecalco-Cruz, 2021; Zhang et al., 2021), with a central role in the antiviral response (Perng and Lenschow, 2018). Previous results reported on the use of ISG15 as adjuvant using different administration doses and routes showed better results with the free-ISG15 form (Villarreal et al., 2015; Iglesias-Guimarais et al., 2020). In this context, we have previously described that the combined expression of HIV-1 antigens with either wild-type or mutated ISG15 delivered from DNA vectors as a prime followed by an MVA-B boost, induced an enhancement of the HIV-1 Env-specific CD8 T cells but not in CD4 T cells. In particular, DNA-ISG15GG showed better immunostimulatory properties than ISG15AA, suggesting a key role for the ISGylation in the connection of the innate and adaptative immune responses (Gómez et al., 2020). In addition to its intracellular role, ISGylated proteins could be released to the extracellular medium where a potential interaction between ISG15 and LFA-1 could occur. LFA-1 is the only ISG15 receptor described to date (Swaim et al., 2017). The role of ISGylated and secreted proteins on the immune response (Swaim et al., 2017; Iglesias-Guimarais et al., 2020) and, more specifically, on the HIV-1-specific CD8 T cells needs to be further defined.

To further characterize the immunomodulatory role of ISG15, in the present work we decided to express ISG15AA and ISG15GG forms from an MVA vector. For this, we generated two different MVA-Δ3-based vectors expressing either ISG15GG or ISG15AA proteins. To know the impact of ISG15 on the immunogenicity elicited against HIV-1 antigens in a mouse model, these vectors were mixed with a recombinant MVA vector expressing HIV-1 Env/Gag-Pol-Nef of clade B (MVA-B). The advantage of the MVA-Δ3-ISG15 vectors is that in addition to its own effect of ISG15 overexpression on HIV-1-specific immune responses, both MVA-based recombinant vectors exhibit a reduced ability to counteract IFN signaling pathways due to the targeted deletion of three VACV immunomodulatory genes, *C6L*, *K7R* and *A46R*, involved in the blocking of IFN-I signalling pathways (García-Arriaza et al., 2014a). The analysis of the viral growth kinetics in permissive (BHK-21) and non-permissive (A549) cell lines confirmed the safety of MVA-based vectors, showing their restriction capacity in human cell lines (Blanchard et al., 1998; Drexler et al., 1998).

As we performed immunization with MVA-based vectors, it is essential to understand what is the impact of the vector during its interaction with the infected cells. Previous studies have demonstrated that MVA is able to activate both Toll-like Receptor (TLR)-dependent and -independent innate immunity pathways in macrophages during infection. Specifically, the TLR2-TLR6 heterodimer receptor is activated in the TLR-dependent pathway, leading to the induction of an immune response that includes T helper type 1 (Th1) cells and NK cells via myeloid differentiation primary response 88 protein (MyD88) interaction, as well as the release of inflammatory cytokines (Zhu et al., 2007; Reed et al., 2009). On the other hand, TLR-independent pathways involves the NALP3 inflammasome, which leads to the production of IL-1β and melanoma differentiation-associated protein 5 (MDA-5), a retinoic acid-inducible gene-I (RIG-I)-like receptor that triggers a cellular feedback mechanism resulting in the release of IFN-I (Waibler et al., 2007). Here, we characterized the effect of ISG15GG or ISG15AA expression in an immortalized bone marrow-derived macrophage cell line, since they play a central role in innate and adaptive immune responses and ISG15 has been reported to regulate macrophage function during viral infections (Yángüez et al., 2013). In this study, using a V5-specific antibody, we detected the presence of heterologous ISG15 both intracellularly and extracellularly after infection, with ISGylation mainly observed following infection with MVA-Δ3-ISG15GG virus. These findings support our previous hypothesis that ISGylated proteins are involved in the activation of the LFA-1 receptor (Swaim et al., 2017). After confirming the proper production of ISG15 in both cell lysates and supernatants of infected cells, we investigated the capacity of the viral vectors to stimulate the production of cytokines involved in the antiviral response. MVA-Δ3-ISG15AA infection *in vitro* and *in vivo* triggers an increase of *Ifn-I* transcription from 6 hours onwards, in correlation with an increase in *Isg15* mRNA levels, and also an increase in soluble IFN-I. At 6 and 16 hours post-infection, MVA-Δ3-ISG15AA produced 4 and 8.5 times more secreted IFN-I than the parental vector, respectively. This increase in IFN level after the infection with the recombinant virus that overexpresses the mutant ISG15AA form (ISGylation deficient), has an impact in the ISGylation, and this phenomenon is more evident *in vivo* that *in vitro*. This could be due to a difference in the timepoints in which we analyzed the ISGylation levels in macrophages (16 hpi) and infected lungs (48 hpi). On the other hand, both systems are completely different, with lung tissue presenting several cell types and an important microenvironment. These features are completely absent in an *in vitro* stabilized cell line which could explain the observed discrepancies.

MVA vector is known to trigger rapid migration of monocytes, neutrophils and CD4 lymphocytes to the inoculation site (Altenburg et al., 2014). Our data indicate a significant increase of IFN-I production in lungs from MVA-Δ3-ISG15AA-infected mice, with no apparent pathological differences between wild-type or mutant ISG15 form. We have previously described that MVA increases IFN levels in infected macrophages (Royo et al., 2014) and DCs (Guerra et al., 2010). The present results indicate that the recombinant MVA expressing the mutant ISG15, unable to perform ISGylation, is a better IFN inducer than both the wild-type ISG15 and parental MVA. Recently, it has been demonstrated using single-cell analysis that MVA infection leads to a division of labor among DCs, with infected DCs producing inflammatory cytokines and non-infected ones secreting T cell costimulatory molecules, indicating that MVA is able to engage both innate and adaptive immunities (Döring et al., 2021). This ability to elicit a robust and specific adaptive immune response, characterized by high affinity and long-term memory, is what makes MVA an excellent vector for vaccination purposes (Burgers et al., 2008; Sánchez-Sampedro et al., 2015).

Another finding on the function of ISG15 is the observation that mutated ISG15 can elicit in mice a higher activation of CD8 T cells specific to HIV-1 antigens (Env/Gag-Pol-Nef) than the wild-type ISG15. The co-administration of MVA-Δ3-ISG15AA with MVA-B in the boost, induced the highest HIV-1 Env- and GPN-specific CD8 T cell response, while for HIV-1 Gag-specific CD8 T cells no differences were observed between the different immunization protocols. The HIV-1-specific CD8 T cells were highly polyfunctional, with cells expressing simultaneously CD107a + IFN-γ + TNF-α being the most representative population, and with the absolute frequency of this population being significantly higher in MVA-Δ3-ISG15AA group. The expression of ISG15 from DNA or MVA vector accounts for the differences observed with our previous work (Gómez et al., 2020), where the wild-type ISG15GG form exhibited the best immunomodulatory effect. In our first immunization schedule (Gómez et al., 2020), DNA-based vectors were primed, and 28 days later, animals were boosted with MVA-WT or MVA-B. In the current study, mice are receiving the boost with the same recombinant virus than in the previous one (MVA-WT or MVA-B), and with the MVA-Δ3-ISG15AA or MVA-Δ3-ISG15GG vectors. As we discussed before, in the *in vivo* system, MVA-Δ3-ISG15AA elicits a higher INF signalling in comparison to MVA-Δ3-ISG15GG, and this could be the reason why in this new immunization regimen MVA-Δ3-ISG15AA is more immunostimulator than the MVA-Δ3-ISG15GG vector. Also, it is probably that, *in vivo*, the enhanced IFN-I stimulation induced by the MVA-Δ3-ISG15AA vector promotes a better expression of MHC-I complex as well as activation of immune cells such as NK, T cells, macrophages and neutrophils, which have a central role in the antiviral response (Perng and Lenschow, 2018; Iglesias-Guimarais et al., 2020).

Overall, our findings are in line with the required features that an ideal adjuvant for HIV-1 vaccine should exert, triggering activation of CD8 T cells and/or NK cells, and avoiding overstimulation of HIV-1-susceptible CD4 T cells (Liu and Ostrowski, 2017). Specifically, MVA-Δ3-ISG15AA virus turned out to exhibit higher immunostimulatory activities than MVA-Δ3-ISG15GG, with the potential effect of ISG15AA as a better adjuvant for its ability to stimulate the production IFN-I. Our results provide support to the importance of ISG15 as an immune adjuvant in the vaccine field and highlights its role as a relevant component in HIV-1 immunization protocols.

## Data availability statement

The original contributions presented in the study are included in the article/supplementary material. Further inquiries can be directed to the corresponding author.

## Ethics statement

The animal study was reviewed and approved by Animal experimental protocols were approved by the Ethical Committee of Animal Experimentation (CEEA) of Centro Nacional de Biotecnología (CNB-CSIC, Madrid, Spain) and of the Universidad Autónoma de Madrid (UAM) according to Spanish National Royal Decree RD 53/2013, Spanish National Law 32/2007 on animal welfare, exploitation, transport and sacrifice and International EU Guidelines 2010/63/UE on protection of animals used for experimentation and other scientific purposes (permit number PROEX 281/16 and PROEX 184.3/22).

## Author contributions

MF, CG, and SG contributed to conception and design of the study. MF, CG, BP, RC, JM, MA, and LM-V performed the experiments. MF, CS, and RC performed the statistical analysis. SG, CG, and ME provided reagents. MF and SG wrote the manuscript. All authors contributed to final manuscript revision, read and approved the submitted version.

## Footnotes

1 Villarreal, D. and Weiner, D. (2019) ‘ISG15 and its use as an adjuvant’. Available at: https://patentSGoogle.com/patent/AU2019202634A1/en (Accessed: 20 November 2022).

2 United Nations Programme on HIV/AIDS (2022) *Global HIV & AIDS statistics — Fact sheet | UNAIDS*. Available at: https://www.unaids.org/en/resources/fact-sheet (Accessed: 17 November 2022).

3 World Health Organization (2022) HIV. Available at: https://www.who.int/news-room/fact-sheets/detail/hiv-aids (Accessed: 17 November 2022).

4 World Health Organization (2022) HIV. Available at: https://www.who.int/news-room/fact-sheets/detail/hiv-aids (Accessed: 17 November 2022).
